# Endoplasmic reticulum stress-related prognosis signature characterizes the immune landscape and predicts the prognosis of colon adenocarcinoma

**DOI:** 10.3389/fgene.2025.1516232

**Published:** 2025-04-01

**Authors:** Lichao Cao, Haoyang Dai, Shangqing Wei, Ying Ba, Fang Chen, Yingying Chen, Chendi Yu, Shenrui Zhang, Erfei Chen, Hezi Zhang

**Affiliations:** ^1^ Key Laboratory of Resource Biology and Biotechnology in Western China, Ministry of Education, Northwest University, Xi’an, China; ^2^ Shenzhen Nucleus Gene Technology Co., Ltd., Shenzhen, China; ^3^ Shanghai Nucleus Biotechnology Co., Ltd., Shanghai, China; ^4^ Department of Research and Development, Shenzhen Nucleus Huaxi Medical Laboratory, Shenzhen, China; ^5^ School of Medicine, Northwest University, Xi’an, China; ^6^ Shaanxi Provincial Key Laboratory of Infection and Immune Diseases, Shaanxi Provincial People’s Hospital, Xi’an, China

**Keywords:** colon adenocarcinoma, endoplasmic reticulum stress, immunotherapeutic sensitivity, prognostic signature, bioinformatics

## Abstract

**Background:**

Colon adenocarcinoma (COAD) is characterized by high mortality and poor prognosis. Endoplasmic reticulum stress-related gene (ERSG) plays an indispensable role in the progression and immunotherapy of COAD. In this study, we evaluated the prognostic value of ERSGs in COAD.

**Methods:**

We constructed and validated the ERSG-related prognostic signature based on public databases using univariate Cox analysis, Kaplan–Meier survival analysis, the LASSO method, and multivariate Cox analysis. In addition, TCGA-COAD, the Human Protein Atlas, and quantitative real-time PCR (q-PCR) were used to detect the mRNA and protein expressions of ERSGs in normal and cancer tissues/cells. The immunotherapeutic cohort was used to evaluate the predictive value of the ERSG signature for immunotherapeutic sensitivity.

**Results:**

The ERSG signature, consisted of HSPA1A, SERPINA1, and DAPK1, could predict the prognosis of patients with COAD. Clinicopathologic characteristics were significantly correlated with risk scores. There were significant differences in the proportion of tumor-infiltrating immune cells, the TP53 mutation rate, the expression of immune checkpoint-related genes, and IC50 of the chemotherapeutic drugs between the low- and high-risk groups. Compared with normal tissues, the mRNA and protein expressions of three ERSGs were decreased in cancer tissues. Compared with NCM460, the mRNA levels of HSPA1A and DAPK1 were decreased in the majority of COAD cell lines, whereas the mRNA level of SERPINA1 was increased in HCT116 and SW480, and reduced in SW620. The ERSG signature could be used as a predictor of immunotherapeutic outcomes.

**Conclusion:**

The ERSG signature has a predictive value in the prognosis and immunotherapeutic sensitivity in COAD, helping guide the personalized treatment.

## 1 Introduction

Colon adenocarcinoma (COAD) is a worldwide gastrointestinal malignancy and is the leading cause of cancerous death. By 2022, there will be more than 1.9 million new cases of colorectal cancer and 904,000 deaths (Freddie et al., 2024). Despite many obvious achievements in improving the prognosis of patients with COAD, such as surgery (Tzu-Chieh et al., 2020), chemotherapy (Bengt et al., 2021), immunotherapy (Ganesh et al., 2019), and radiotherapy (Hao et al., 2020), a proportion of patients with COAD unfortunately cannot benefit from these therapies. Therefore, clarifying the molecular mechanism and finding new therapeutic targets is an urgent need to effectively ameliorate or even significantly facilitate COAD therapy.

The endoplasmic reticulum is responsible for protein synthesis, processing, and transportation (Paolo and Suzanne, 2008; Kapuy, 2024). Studies have shown that oxidative stress, nutrient deficiency, hypoxia, ischemia, and DNA damage in the tumor microenvironment (TME) disrupted the homeostasis of the endoplasmic reticulum, resulting in abnormal accumulation of misfolded or unfolded proteins, thus inducing endoplasmic reticulum stress (Scott A and Feroz R, 2014; Hanna et al., 2014). In addition, endoplasmic reticulum stress can dynamically reprogram the function of immune cells, thus facilitating immune escape of tumor cells (Cubillos-Ruiz et al., 2017; Yuan et al., 2022). Conversely, endoplasmic reticulum stress can significantly induce apoptosis of tumor cells and strengthen the efficacy of anticancer drug therapy by mediating the pro-death pathway (Lindner et al., 2020; Nami et al., 2016). Importantly, PERK (an endoplasmic reticulum stress inducer) activator CCT020312 promoted PERK activation to improve the efficacy of Taxol in colorectal cancer (Lei et al., 2021).

To investigate the endoplasmic reticulum stress on the prognosis of COAD, we used prognostic endoplasmic reticulum stress-related genes (ERSGs) to construct an ERSG signature for COAD. We also explored survival analysis, the tumor immune microenvironment, somatic mutation, nomogram construction, prediction of immune response, and chemotherapy drugs based on risk signatures. Generally, our study offered a reference value for exploring the progression and immunotherapy of COAD from the perspective of endoplasmic reticulum stress.

## 2 Materials and methods

### 2.1 Data source and process

ERSGs with a correlation score ≥5 were obtained from the GeneCards database (https://www.genecards.org/). The RNA-seq transcriptome, survival information, clinical information, and somatic mutation data on the COAD samples (432 tumor datasets) and the normal samples (39 normal datasets) were obtained from the UCSC Xena platform (https://xenabrowser.net/datapages/). Additionally, the GSE39582 dataset from the Gene Expression Omnibus (GEO) (https://www.ncbi.nlm.nih.gov/geo/) database, which contains 556 COAD samples with survival and clinical information, was used as an external validation cohort. GSE200997, a single-cell next-generation RNA sequencing (scRNA-seq) dataset, was used to analyze the expression of the candidate prognostic ERSGs in the scRNA-seq dataset. The analysis process of scRNA-seq dataset GSE200997 was consistent with that of our previous study (Cao et al., 2023). In addition, an immunotherapy cohort (anti-PD-L1 in IMvigor210 cohort) was obtained from a published study (Mariathasan et al., 2018), containing the mRNA expression profile and clinical data. The samples were divided into platinum-treated cohorts (N = 105, receiving platinum-based chemotherapy) and non-platinum-treated cohorts (N = 237, not receiving platinum-based chemotherapy).

### 2.2 Establishment and validation of an ERSG signature

To acquire ERSGs with prognostic abilities, the univariate Cox analysis was used to initially filter prognosis-related ERSGs with *P* < 0.05. Subsequently, the Kaplan–Meier survival analysis further screened for prognosis-related ERSGs (*P* < 0.05). Last, the LASSO regression analysis was used to screen out optimal prognostic ERSGs with | coefficients| > 0.1 using the *glmnet* R package. The multivariate Cox analysis was used to construct a prognostic signature using the *glmnet* R package and offer the coefficient of ERSGs. Based on the expression and coefficient of prognosis-related ERSGs, the following equation was used to calculate the risk score:

$$risk\;score=\sum_{i}^{n}\left({Cox\;coefficient\;of\;x}_{i}\times {scaled\;expression\;value\;of\;x}_{i}\right),$$

where χi indicates the current biomarker (gene), i is the location of the current biomarker, and n represents the number of biomarkers.

*SurvivalROC* in the R package was used to quantify the area under the curve (AUC) of the receiver operating characteristic (ROC) curve. The ROC curve with the most significant difference between true positive and false positive was selected, and the turning point of the curve was selected as the best cutoff value, according to which patients were allocated into high- and low-risk groups. To assess the predictive efficiency of the ERSG signature, Kaplan–Meier survival analysis was used to distinguish survival differences between low- and high-risk groups using *survminer* in the R package.

To verify the applicability of the model, the GSE39582 cohort was served as an independent external validation set. Similarly, according to the best cutoff value of the ROC curve, patients were divided into the low- and high-risk groups. Kaplan–Meier survival analysis and ROC curves were plotted, respectively.

### 2.3 Association of the ERSG signature with clinicopathological traits

Kruskal–Wallis or Wilcoxon tests were used to investigate the correlation between the ERSG signature and the clinicopathological traits (including advanced pathological stages, and T, N, and M stage traits) in TCGA-COAD and GSE39582 cohorts, respectively.

### 2.4 Nomogram and calibration analysis

The nomogram, including the risk score, age, gender, the microsatellite status, and the tumor stage, was created to forecast the overall survival (OS) of 1, 3, and 5 years using the *rms* R package. The calibration curve was used to assess the predicted consistency of the nomogram.

### 2.5 Analysis of immune infiltration and somatic mutation

The proportion of 22 kinds of tumor-infiltrating immune cells was calculated using the CIBERSORT algorithm. Subsequently, the unpaired *t*-test was used to estimate the comparison of the immune landscape between high- and low-risk groups in the TCGA-COAD cohort. The *maftools* R package was employed to calculate and visualize the mutation profiles and the tumor mutation burden (TMB) value of the high- and low-risk groups in the TCGA-COAD cohort. Kaplan–Meier survival analysis was employed to measure the correlations of gene mutation status with OS. The unpaired *t*-test was employed to compare the TMB values between the high- and low-risk groups in the TCGA-COAD cohort. The relationship among TMB values, TMB values combined risk scores, and OS was assessed using Kaplan–Meier curves.

### 2.6 Immune checkpoint and chemotherapeutic reaction analyses

The unpaired *t*-test was conducted to compare the mRNA levels of immune checkpoint-related genes (such as CD274, PDCD1, CTLA4, HAVCR2, LAG3, and TIGIT) in the two groups in the TCGA-COAD cohort. Moreover, the Wilcoxon test was used to calculate the differences in eight chemotherapy drugs’ IC50 (half-maximal inhibitory concentration) in the low- and high-risk groups in the TCGA-COAD cohort.

### 2.7 Molecular docking analysis

The UniProt bank (https://www.uniprot.org/) provided the structures of target proteins (HSPA1A, SERPINA1, and DAPK1). The PubChem website (https://pubchem.ncbi.nlm.nih.gov/) provided the structures of the drug chemical formula. ‘.sdf’ files were converted into a unified ‘.pdb’ format using Open Babel GUI software. Finally, DeepMice (http://www.deepmice.com/) was used to perform molecular docking, and the results were visualized.

### 2.8 Comparison of the ERSG expression in normal and COAD tissues

The unpaired *t*-test was conducted to assess the comparison of ERSG mRNA expressions between normal and COAD tissues in the TCGA-COAD cohort. Furthermore, we examined the protein expressions of ERSGs in normal colon and COAD tissues using the immunohistochemical images from the Human Protein Atlas (HPA; https://www.proteinatlas.org/).

### 2.9 Cell culture and quantitative real-time PCR

We obtained human colorectal cancer cells (HT-29, HCT116, SW480, and SW620) from the American Type Culture Collection and immortal normal epithelial cells (NCM460) from INCELL (San Antonio). These cells were grown in a prescribed McCoy’s 5A, Leibovitz’s L-15, or RPMI-1640 medium with 10% fetal bovine serum (Gibco Company) at 37°C and 5% CO_2_ in a cell incubator. Total cellular RNA was extracted using TRIzol reagent (#R401-01, Vazyme) based on standard protocols. Then, the obtained RNAs were reverse-transcribed to cDNA using the HiScript II 1st Strand cDNA Synthesis Kit (#R211-01, Vazyme). Subsequently, q-PCR was performed with the AceQ Universal SYBR qPCR Master Mix (#Q111-02, Vazyme), and the relative quantitative value was calculated using the 2^−ΔΔCT^ method. The primer sequences are shown in Supplementary Table S1.

### 2.10 The role of ERSG signature in immunotherapy

The platinum-treated and non-treated datasets from the IMvigor210 cohort were used to validate the constructed ERSG signature. Then, patients were divided into responders [patients with complete response (CR) or partial response (PR)] and non-responders [patients with stable disease (SD) or progressive disease (PD)], and the risk score was calculated based on the built ERSG signature. Furthermore, the Wilcoxon test was used to compare the tumor mutation burden and neoantigen burden between two risk groups.

### 2.11 Statistical analysis

GraphPad Prism 8.0 software was used to perform statistical analysis. Student’s t-test was used to assess the differences between the two groups. Data were expressed as the mean ± standard deviations. *P* < 0.05 was set as the significance level.

## 3 Results

### 3.1 Construction and validation of the ERSG prognostic risk model

**FIGURE 1:**
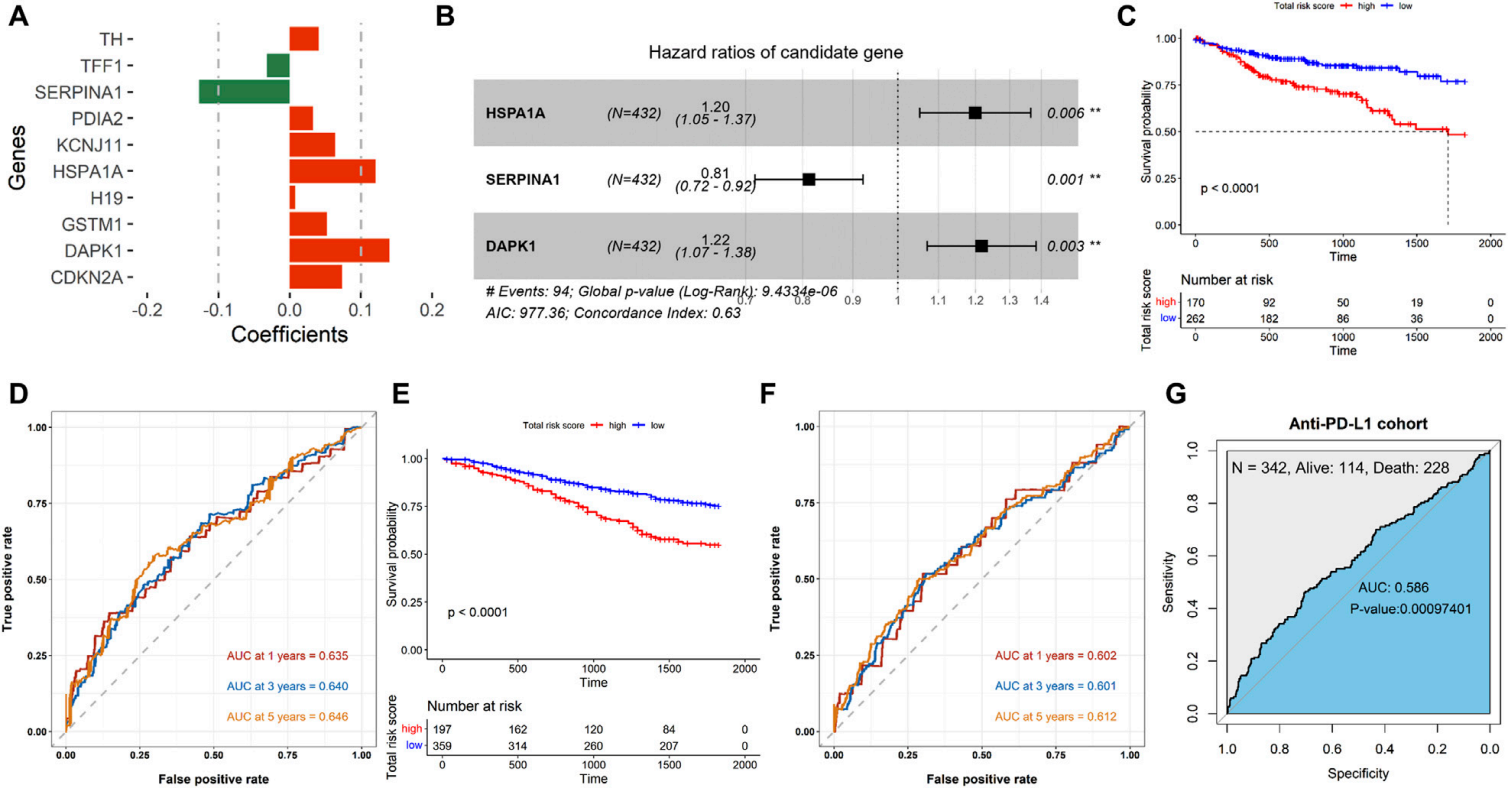
Establishment and validation of the ERSG signature. **(A)** Coefficient information on the ERSGs obtained using LASSO regression analysis. **(B)** Forest map of multivariate Cox analysis. Kaplan–Meier survival analyses of the ERSG signature in the **(C)** TCGA-COAD and **(E)** GSE39582 cohorts. ROC survival of the ERSG signature in the **(D)** TCGA-COAD, **(F)** GSE39582, and **(G)** anti-PD-L1 cohorts.

A total of 1,181 ERSGs were obtained from the GeneCards database. Based on the TCGA-COAD cohort, we first filtered 41 ERSGs with a prognostic value using univariate Cox analysis (*P* < 0.05, Supplementary Table S2). The Kaplan–Meier survival analysis further screened 17 ERSGs associated with prognostic significance (*P* < 0.05, Supplementary Figures S1, 2). We also performed a LASSO regression analysis with |coefficients| >0.1, and three ERSGs were finally identified as optimal prognostic signatures (Figure 1A), namely, HSPA1A, SERPINA1, and DAPK1. Accordingly, multivariate Cox analysis constructed an ERSG signature based on the three prognostic factors (Figure 1B). The ERSG-related risk score was calculated as follows: risk score = [0.1816732 × mRNA expression of HSPA1A] + [-0.2082352× mRNA expression of SERPINA1] + [0.1965172 × mRNA expression of DAPK1]. The patients were divided into high- and low-risk groups according to the cutoff value of ROC curves. The Kaplan–Meier survival analysis demonstrated that patients in the low-risk group had a better OS (*P* < 0.0001, Figure 1C). Next, we used the ROC curve to evaluate the accuracy of three ERSGs in predicting the prognosis of COAD. The AUC of the three ERSGs was 0.635 at 1 year, 0.640 at 3 years, and 0.646 at 5 years (Figure 1D). In addition, the GSE39582 dataset was employed for validation. As demonstrated, patients from the high-risk group had a worse prognosis (*P* < 0.0001, Figure 1E). Analogously, the 1-, 3-, and 5-year AUCs of the ROC curve were greater than 0.6 (Figure 1F). Furthermore, the AUC of the anti-PD-L1 cohort was 0.586 (*P* = 0.00097401, Figure 1G). These results suggest that the risk score can be used to predict the prognosis of patients with COAD and provide a reference for predicting immunotherapy in COAD patients.

### 3.2 Relationship of the ERSG signature with clinical traits

**FIGURE 2:**
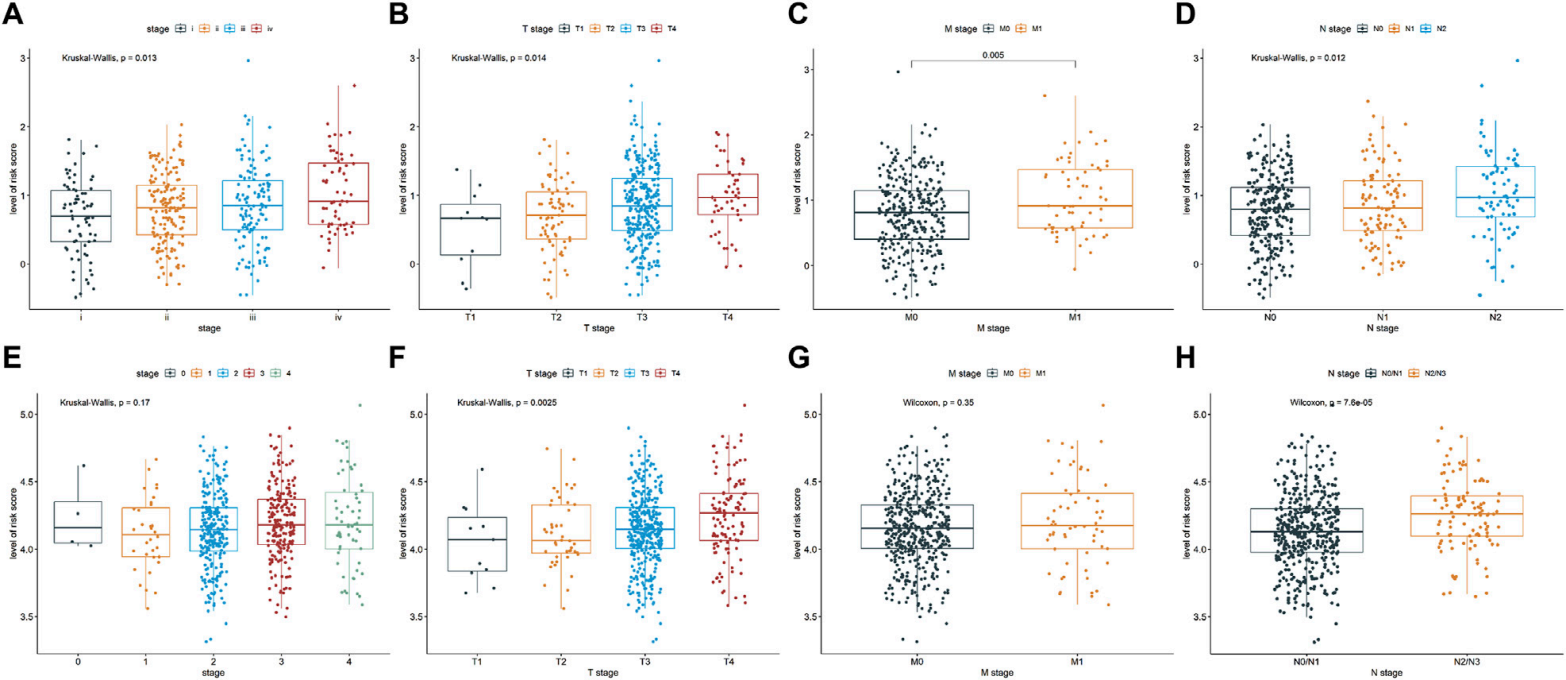
Relationship of the ERSG signature with clinicopathological characteristics, such as pathological stage **(A)** TCGA-COAD cohort, n = 421; **(E)** GSE39582 cohort, n = 556), T stage **(B)** TCGA-COAD cohort, n = 431; **(F)** GSE39582 cohort, n = 532), M stage **(C)** TCGA-COAD cohort, n = 378; **(G)** GSE39582 cohort, n = 534), and N stage **(D)** TCGA-COAD cohort, n = 432; **(H)** GSE39582 cohort, n = 530).

As demonstrated in Figures 2A–D, there were significant differences in the pathological stage (*P* = 0.013), T stage (*P* = 0.014), M stage (*P* = 0.005), and N stage (*P* = 0.012) between high- and low-risk groups in the TCGA-COAD cohort. Moreover, the high-risk group exhibited late T stage (*P* = 0.0025, Figure 2F) and late N stage (*P* = 7.6e-05, Figure 2H) in the GSE39582 cohort, whereas no significant differences were observed in the pathological stage (*P* = 0.17, Figure 2E) and M stage (*P* = 0.35, Figure 2G) between two groups. These findings indicated that the risk score is closely related to the clinicopathological traits of COAD patients.

### 3.3 Nomograph construction

**FIGURE 3:**
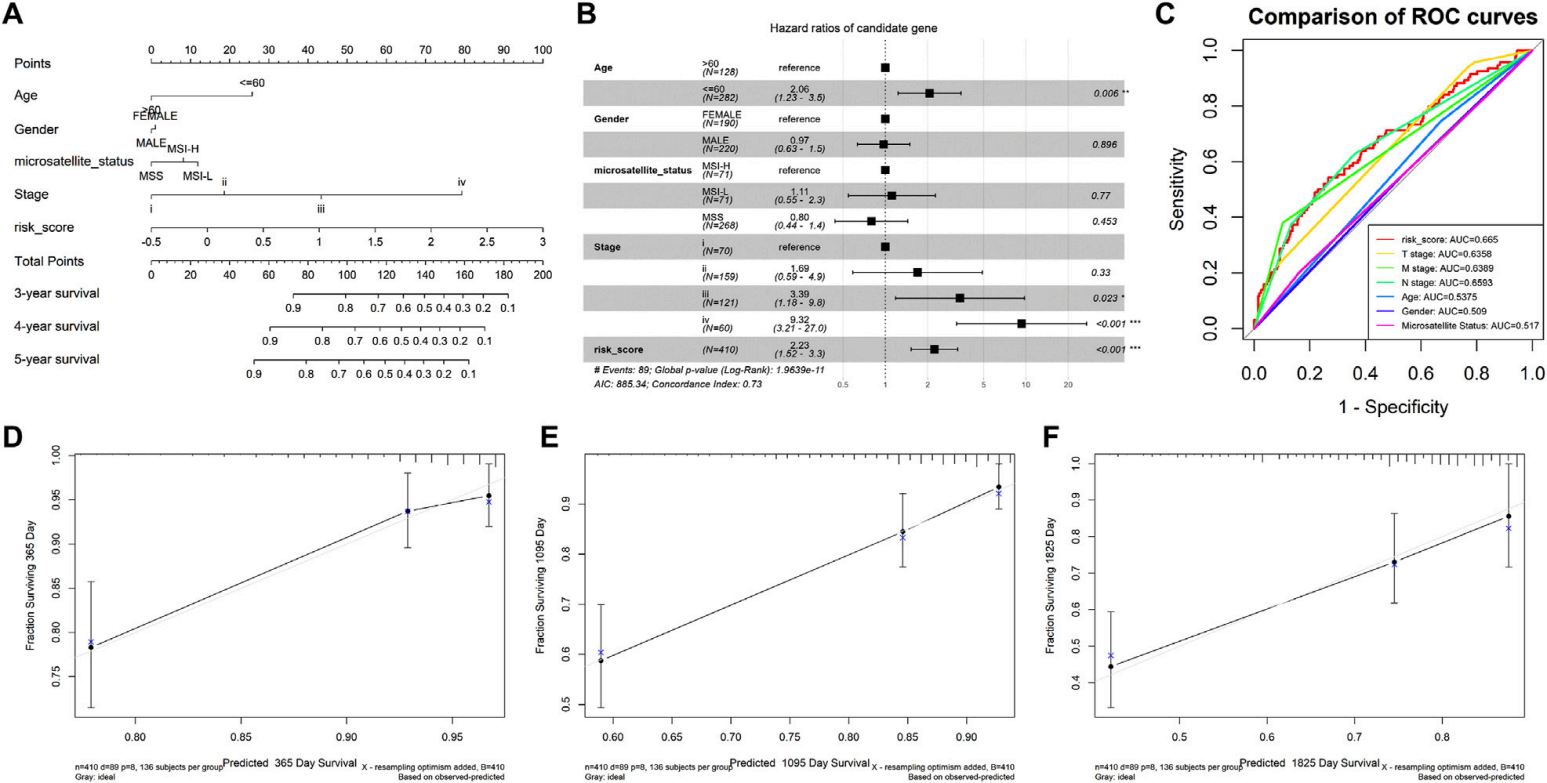
Nomogram construction in TCGA-COAD. **(A)** Nomogram was drawn to predict the survival of the model. **(B)** Hazard ratios of the clinical traits and risk score. **(C)** Comparison of ROC curves between the risk score and other clinical parameters. Calibration curve of 1 year **(D)**, 3 years **(E)**, and 5 years **(F)**.

Next, we constructed a nomographic chart to quantitatively predict the prognosis of the COAD patients using age, sex, microsatellite status, pathological stage, and risk score (Figure 3A). Furthermore, the forest plot demonstrated that the risk score, age (≤60), and stage IV were independent prognostic factors (*P* < 0.05, Figure 3B). In addition, the ROC curve indicated that the risk score has the largest AUC value (AUC = 0.665), suggesting that the risk score has stronger predictive power than other clinical parameters (Figure 3C). The calibration curves indicated that the predictive curves of 1 year, 3 years, and 5 years almost coincide with the ideal curves (Figures 3D–F), suggesting that the clinical nomogram has good ability in predicting the prognosis of COAD.

### 3.4 Analysis of immune infiltration and mutation profiles

**FIGURE 4:**
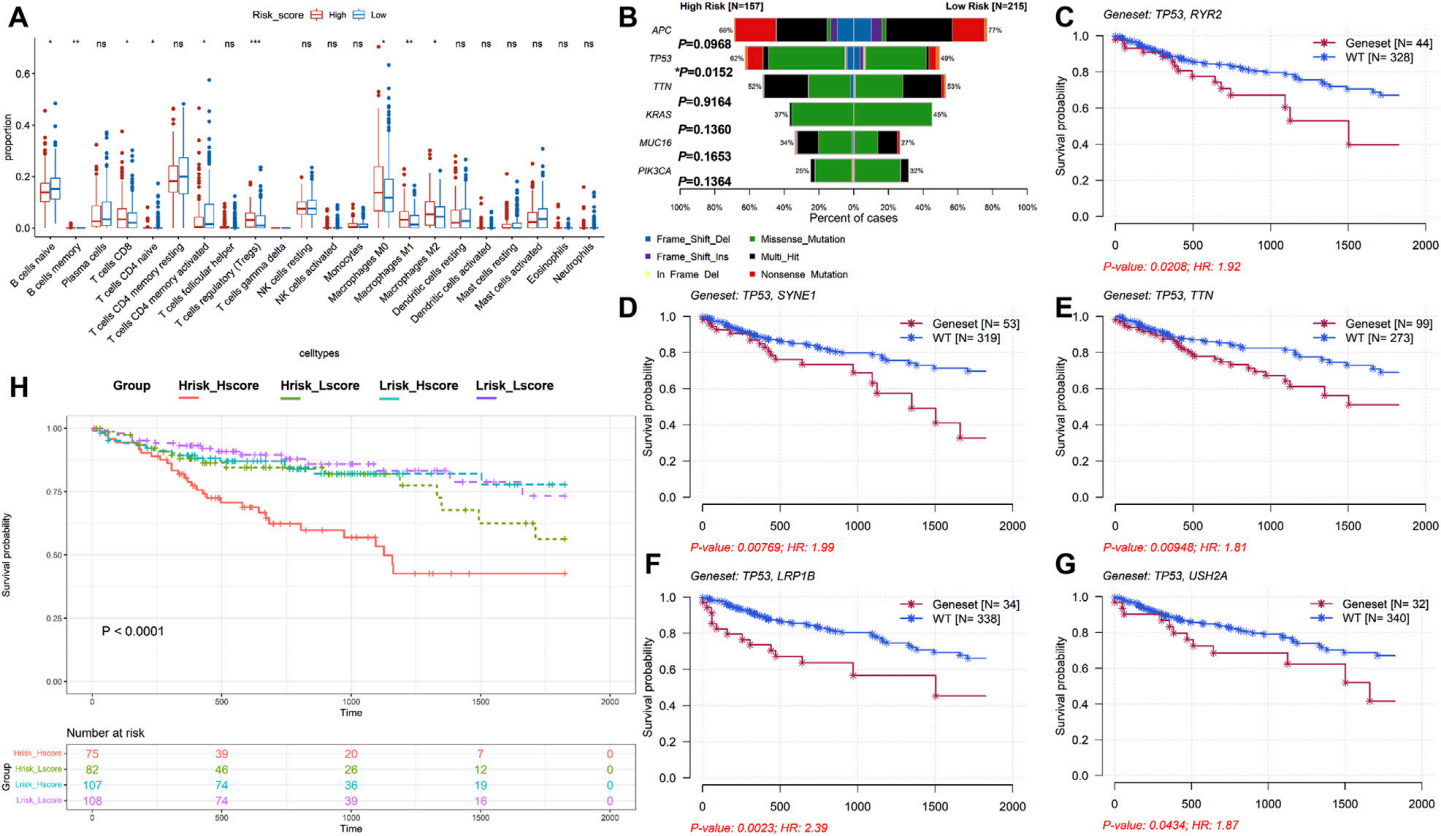
Analysis of immune cell infiltration and mutation profiles in TCGA-COAD. **(A)** Immune cell infiltration analysis. High-risk group: 167; low-risk group: 254; ^ns^ *P* > 0.05, *P < 0.05, **P < 0.01, and ***P < 0.001. **(B)** Comparison of the mutation rate between high- and low-risk groups. **(C–G)** Kaplan–Meier survival analyses of the mutation status of the combined TP53 with other genes. **(H)** Kaplan–Meier survival analysis of the combined TMB-values with the risk score.

The level of infiltration of immune cells in the tumor microenvironment plays a critical role in prognosis and efficacy of cancer. According to the box plot, cells such as naïve B cells, memory B cells, CD8^+^ T cells, naïve CD4^+^ T cells, activated memory CD4^+^ T cells, T-cell regulatory (Tregs), and M0/M1/M2 macrophages were all sensitive to the risk score (*P* < 0.05, Figure 4A). Among them, only naïve B cells, naïve CD4^+^ T cells, and activated memory CD4^+^ T cells were increased in the low-risk group. Moreover, we analyzed somatic mutations in each patient using the TCGA-COAD cohort. The top five observably mutated genes were APC, TP53, TTN, KRAS, and MUC16 in the high-risk group (Supplementary Figure S3A, left), whereas those in the low-risk group were APC, TTN, TP53, KRAS, and PIK3CA (Supplementary Figure S3A, right). Among them, the mutation rate of TP53 was higher in the high-risk group (*P* = 0.0152, Figure 4B). Surprisingly, the mutation status of TP53 did not affect the survival time of patients (*P* = 0.183, Supplementary Figure S3B). However, co-mutation of TP53 and other genes, such as RYR2 (*P* = 0.0208), SYNE1 (*P* = 0.00769), TTN (*P* = 0.00948), LRP1B (*P* = 0.0023), and USH2A (*P* = 0.0434), had a worse prognosis (Figures 4C–G). Continuously, we examined the TMB values in the TCGA-COAD cohort. The results showed that a lower TMB value was discovered in the high-risk group (2.18/MB vs. 2.22/MB) (Supplementary Figure S3C), whereas the difference was not statistically significant (*P* = 0.54, Supplementary Figure S3D). Meanwhile, the TMB values were not associated with prognosis (*P* = 0.057, Supplementary Figure S3E). However, combined TMB values and risk-score grouping could predict the prognosis of COAD (*P* < 0.0001, Figure 4H).

### 3.5 Immune checkpoint and chemotherapeutic reaction analyses

**FIGURE 5:**
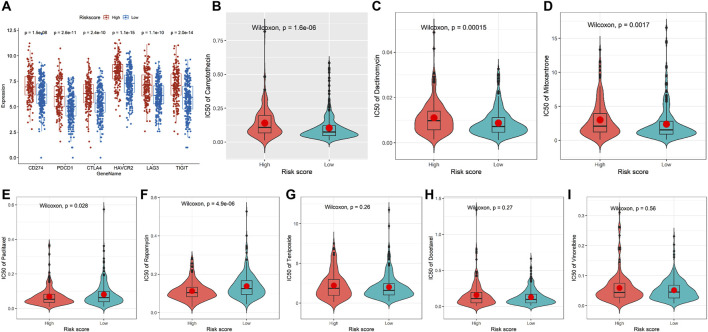
Immune checkpoint and chemotherapeutic reaction analyses in TCGA-COAD. **(A)** Comparison of the mRNA levels of immune checkpoints and their ligands in the two groups. **(B–I)** Comparison of the IC50 values of chemotherapy drugs in the two groups. High-risk group: 167 and low-risk group: 254.

**FIGURE 6:**
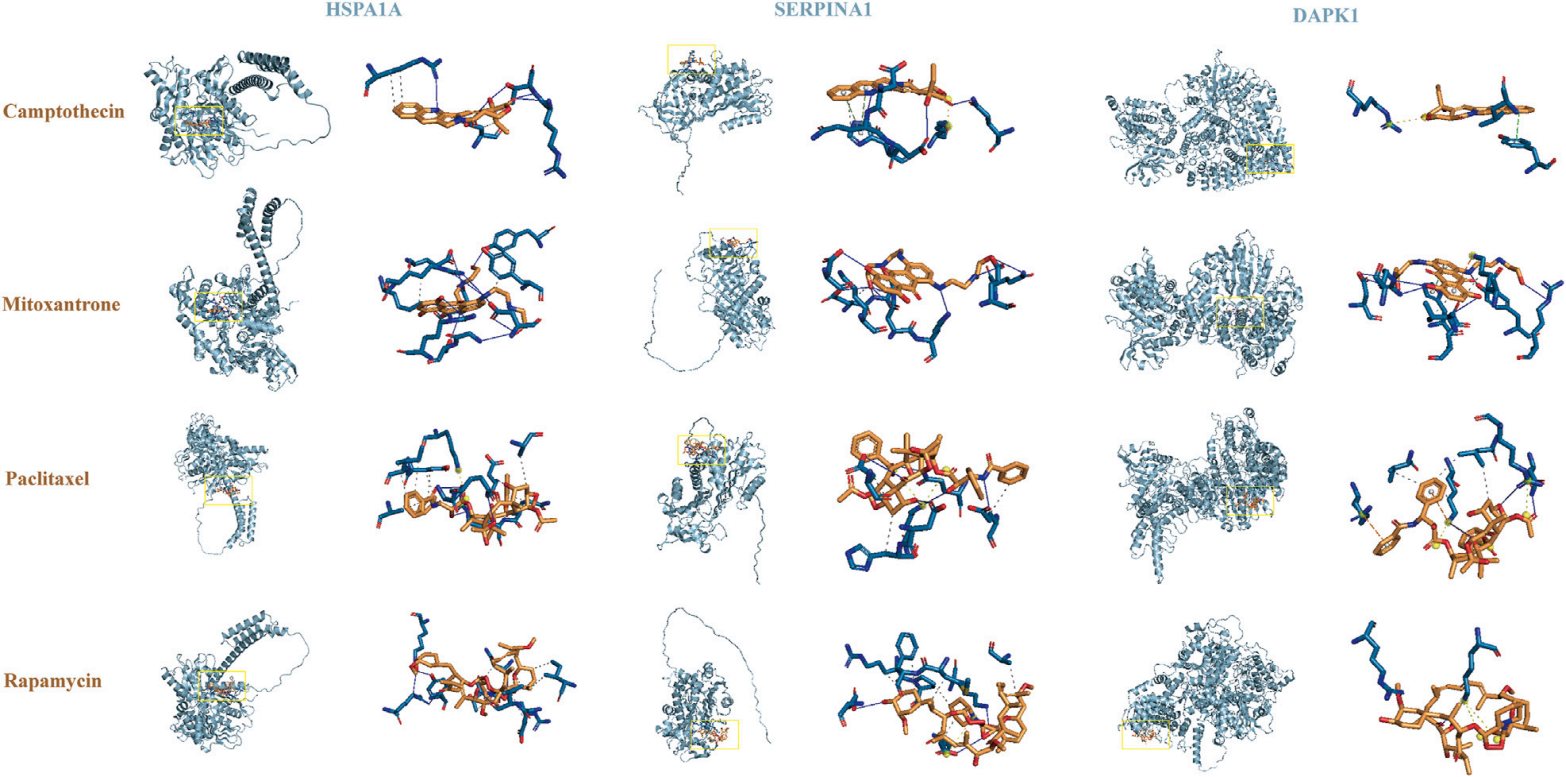
Molecular docking analysis.

The results suggested that patients with high-risk scores had higher levels of CD274, PDCD1, CTLA4, HAVCR2, LAG3, and TIGIT expression, indicating that these patients may benefit from immune checkpoint inhibitor therapy (Figure 5A). In addition, compared with the low-risk group, the IC50 values of camptothecin (*P* = 1.6e-06), dactinomycin (*P* = 0.00015), and mitoxantrone (*P* = 0.0017) were increased and the IC50 values of paclitaxel (*P* = 0.028) and rapamycin (*P* = 4.9e-06) were decreased in the high-risk group (Figures 5B–F). However, there was no significant difference in the IC50 values of teniposide (*P* = 0.26), docetaxel (*P* = 0.27), and vinorelbine (*P* = 0.56) between the two groups (Figures 5G–I). Subsequently, we further analyzed the interactions between identified target proteins (HSPA1A, SERPINA1, and DAPK1) and chemotherapy drugs (camptothecin, mitoxantrone, paclitaxel, and rapamycin) of COAD using molecular docking techniques. The docking results showed that HSPA1A has the best binding affinity with mitoxantrone, SERPINA1 has the best binding affinity with mitoxantrone, and DAPK1 has the best binding affinity with paclitaxel (Table 1; Figure 6). These findings have a predictive value for clinical treatment and medication of COAD patients.

**TABLE 1:** Information on molecular docking.

Drug_PubChem_ID	Drug name	Gene name	UniProt	Docking score	Number of hydrophobic interactions	Number of hydrogen bonds
24,360	Camptothecin	HSPA1A	P0DMV8	3.758	4	5
4,212	Mitoxantrone			18.851	2	12
36,314	Paclitaxel			1.870	10	3
5284616	Rapamycin			2.118	7	6
24,360	Camptothecin	SERPINA1	P01009	1.776	2	3
4,212	Mitoxantrone			3.584	2	9
36,314	Paclitaxel			2.668	3	7
5284616	Rapamycin			2.232	6	4
24,360	Camptothecin	DAPK1	P53355	3.407	1	0
4,212	Mitoxantrone			5.183	2	7
36,314	Paclitaxel			5.540	4	3
5284616	Rapamycin			4.526	0	2

### 3.6 Evaluation of the ERSG expression in normal and COAD tissues, cell lines, and the scRNA-seq dataset

**FIGURE 7:**
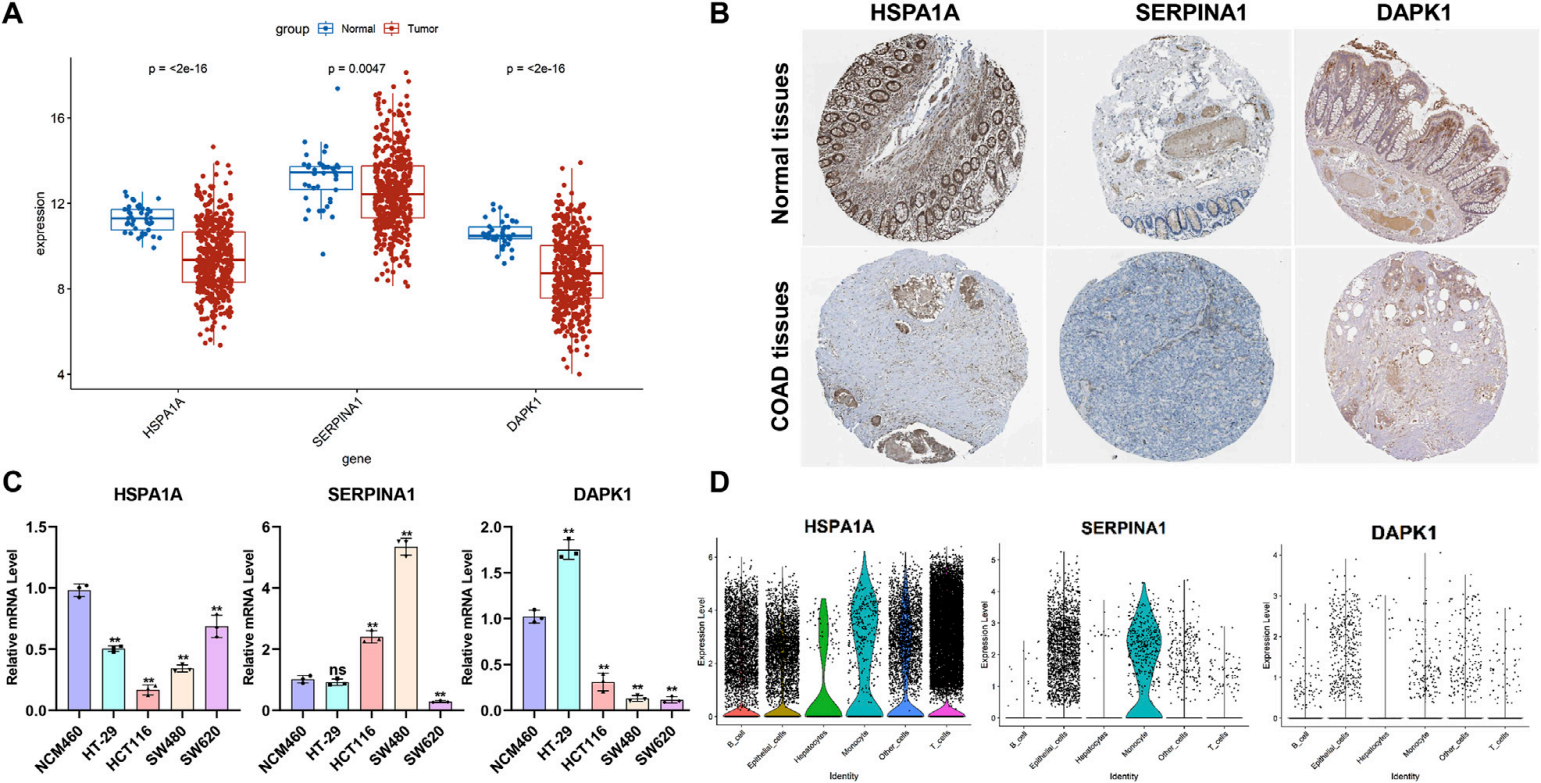
Analysis of the expression levels of the ERSGs in COAD and scRNA-seq. **(A)** mRNA data from the TCGA COAD cohort. Normal: 39 and tumor: 432. **(B)** Immunohistochemical images from the HPA database. **(C)** q-PCR was used to analyze the mRNA level of three ERSGs in normal epithelial cells (NCM460) and colorectal cancer cells (n = 3, ^ns^ *P* > 0.05, *P < 0.05). **(D)** Expression level of ERSGs in each cell types based on scRNA-seq dataset GSE200997.

To further evaluate the ERSG expression, we used the TCGA-COAD cohort and the HPA database to compare the mRNA and protein expression of three ERSGs in normal and COAD tissues, respectively. As shown in Figure 7A, compared with normal tissues, the mRNA levels of HSPA1A, SERPINA1, and DAPK1 were decreased in COAD tissues. Moreover, the immunohistochemical results of the HPA database indicated that protein expressions of HSPA1A, SERPINA1, and DAPK1 were downregulated in COAD tissues compared with normal colon tissues (Figure 7B; Supplementary Table S3). Compared to NCM460, the mRNAs of HSPA1A and DAPK1 were significantly decreased in the majority of COAD cell lines, and the mRNA of SERPINA1 was significantly increased in HCT116 and SW480, and decreased in SW620 (Figure 7C). In scRNA-seq dataset GSE200997, HSPA1A was widely distributed in various cell types, and SERPINA1 was mainly expressed in endothelial cells and monocytes, whereas DAPK1 was less distributed in all kinds of cells (Figure 7D). These findings suggested that HSPA1A, SERPINA1, and DAPK1 are involved in the pathogenesis and metastasis.

### 3.7 Evaluation of the immunotherapeutic response in the ERSG signature

**FIGURE 8:**
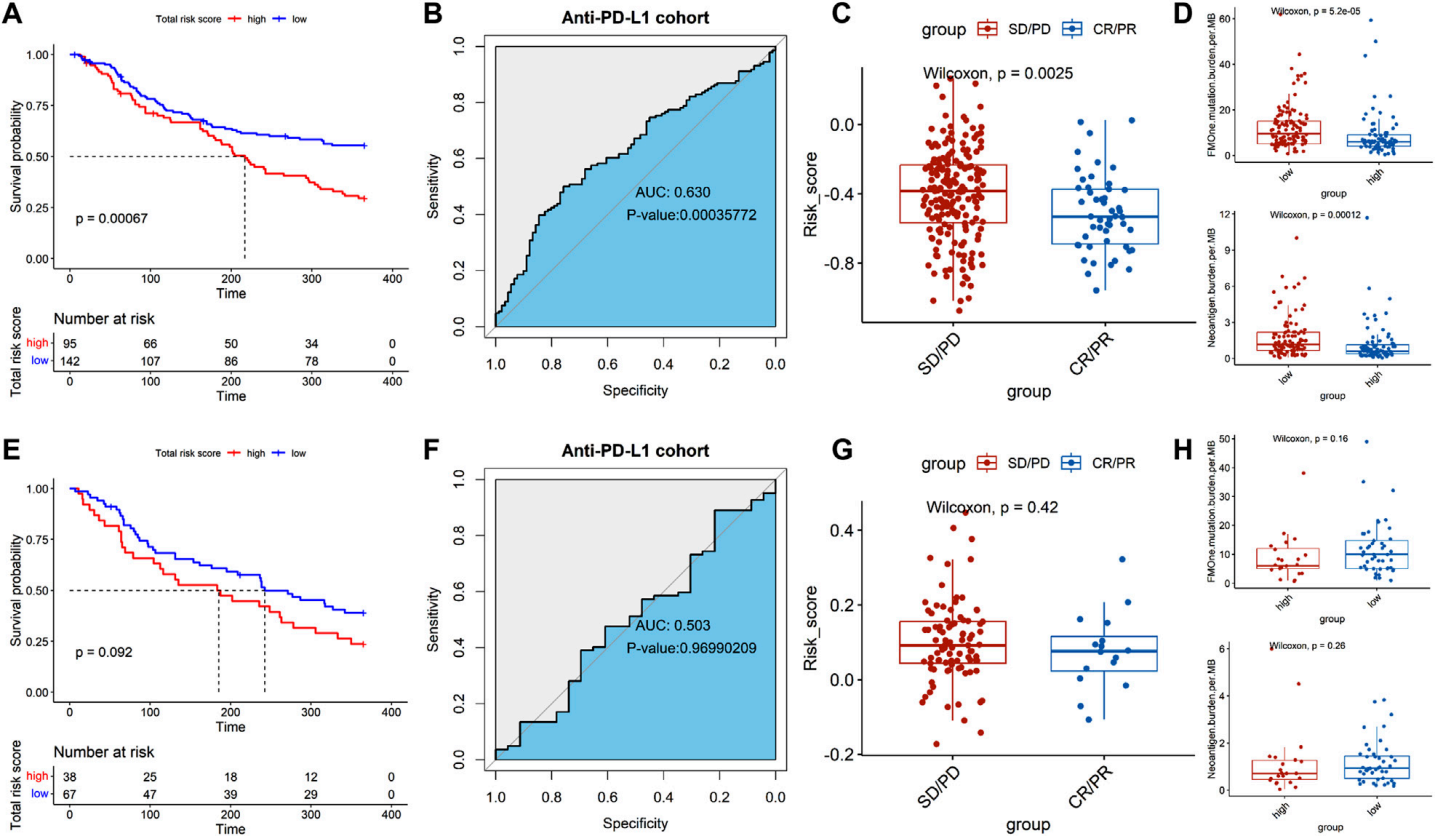
Role of the ERSG signature in the IMvigor210 cohort. The correlation of the risk score with OS in **(A)** non-platinum-treated and **(E)** platinum-treated cohorts. ROC survival of the ERSG signature in the anti-PD-L1 cohort from the **(B)** non-platinum-treated and **(F)** platinum-treated cohorts. Comparison of the risk score between the non-responder and responder groups in **(C)** non-platinum-treated [SD/PD: 40/107, CR/PR: 18/32] and **(G)** platinum-treated cohorts [SD/PD: 22/57, CR/PR:6/10]. Comparison of the tumor mutation burden and neoantigen burden between high- and low-risk groups in **(D)** non-platinum-treated [high-risk group: 95 and low-risk group: 142] and **(H)** platinum-treated cohorts [high-risk group: 38 and low-risk group: 67].

To verify whether the ERSG signature can be used as a tool to assess the immunotherapeutic response, the urothelial cancer cohort receiving anti-PD-L1 agent treatment (atezolizumab for IMvigor210) was used for validation. The results showed that the non-platinum-treated patients with high-risk scores suffered poor prognoses (*P* = 0.00067, Figure 8A), and the ERSG signature provided a reference for predicting immunotherapeutic effectiveness in the non-platinum-treated cohort (AUC = 0.630, *P* = 0.00035722, Figure 8B). Meanwhile, the non-responder group had a higher risk score than the responder group (*P* = 0.0025, Figure 8C). Furthermore, higher tumor mutation load (*P* = 5.2e-05) and neoantigen burden (*P* = 0.00012) in non-platinum-treated patients were significantly associated with high-risk scores (Figure 8D). However, the ERSG signature cannot be used as a tool to assess the immunotherapeutic response in platinum-treated patients (Figures 8E–H).

## 4 Discussion

COAD is a common malignant tumor of the digestive tract, with high morbidity and mortality (Wang H. et al., 2023). Studies have shown that the abnormal state of the TME can cause persistent stress of the endoplasmic reticulum, thereby dynamically reprogramming the function of immune cells and activating the pro-tumor signals of cancer cells (Chen and Cubillos-Ruiz, 2021). Currently, most studies focus on the effects of endoplasmic reticulum stress on cancer biological activity, whereas only a few studies focus on the prognostic significance of ERSGs, especially COAD. In this study, we established a prognostic ERSG signature for COAD to systematically analyze the relationship among ERSG signatures, the TME, and immunotherapy efficacy, offering critical insights for improving the precision therapy of COAD.

At present, some studies have focused on the prognostic value of ERSGs in COAD; however, the number of prognostic genes constructed in such prognostic models is large (Yang et al., 2022; Liu et al., 2022; Chen X. et al., 2022; Geng et al., 2023). Here, establishment of the prognostic model identified three novel ERSGs with the prognostic value, including HSPA1A, SERPINA1, and DAPK1. HSPA1A, as a member of the heat shock protein 70 (HSP70) family, is a protective protein with elevated expression after stress (He et al., 2023) and assists in protein folding (Finka et al., 2015). When the homeostasis of the endoplasmic reticulum changes, it leads to abnormal accumulation of misfolded or unfolded proteins, inducing endoplasmic reticulum stress (Lam et al., 2020). In this stress states, the expression of the HSP70 family is increased, thereby initiating a protective mechanism against endoplasmic reticulum stress (Chen W. et al., 2022). In addition, HSPA1A is overexpressed in many types of cancers and helps cancer cells proliferate (Daugaard et al., 2005). Additionally, HSPA1A is considered a therapeutic target for cancer therapy (Shevtsov et al., 2018). However, Guan et al. (2021) demonstrated that HSPA1A was significantly downregulated in colon cancer tissues compared with adjacent non-tumor tissues, and high expression of HSPA1A had a worse overall survival, which is consistent with our results. These findings suggested that HSPA1A contributes to the development and poor prognosis for COAD. SERPINA1 belongs to the serine protease inhibitor family, and its mutations cause accumulation of misfolded alpha-1 antitrypsin during endoplasmic reticulum stress (Greene et al., 2016). SERPINA1 is abnormally expressed in multiple cancers, such as skin squamous cell carcinoma (Farshchian et al., 2011), breast cancer (Abbott et al., 2008; López-Árias et al., 2012), and lung cancer (Topic et al., 2011). Moreover, an increase in SERPINA1 was related to advanced stage and poor prognosis of colorectal cancer, being considered a reliable prognostic biomarker and therapeutic target in colorectal cancer (Kwon et al., 2015). DAPK1 acts as death-associated protein kinase 1, playing an important role in tumor suppression by mediating the endoplasmic reticulum stress-dependent apoptotic pathway (Wang et al., 2017; Raveh et al., 2001; Shanmugam et al., 2012). Moreover, ATF6 (an endoplasmic reticulum-resident factor)/DAPK1-mediated mAtg9a trafficking triggered endoplasmic reticulum stress-induced autophagy (Zhou et al., 2016). Yuan et al. (2017) considered that the methylation of DAPK1 had an ability in diagnosing for early gastrointestinal cancer. In colorectal cancer, downregulation of DAPK1 was associated with poor prognosis of patients (Hsin-Yi et al., 2012). Similarly, our results showed the mRNA of DAPK1 was increased in HT-29 (a type of early-stage colorectal cancer cell) and reduced in advanced-stage cell lines, such as HCT116, SW480, and SW620, indicating that the downregulation of DAPK1 has a connection with COAD progression. This finding demonstrated that three ERSGs can serve as potential predictive biomarkers for COAD, suggesting that ERSGs were associated with the progression of COAD.

Subsequently, we successfully established a reliable ERSG-related risk model for COAD. Consistent with other similar models (Zhang et al., 2023; Wang Y. et al., 2023), our risk signature had a strong correlation with OS and showed moderate performance in prognosis prediction. In addition, our risk signature demonstrated that patients with lower risk scores showed better prognoses and served as an independent prognostic factor. Meanwhile, the constructed nomograph exhibited high accuracy in the prediction of the 1-, 3-, and 5-year survival.

Given that previous studies have suggested the prominent role of immune-related activities in the progression of COAD (Picard et al., 2020; Yao et al., 2022), we further analyzed the differences in immune-infiltrating cells between low-risk and high-risk groups. From CIBERSORT results, the proportion of naïve B cells, naïve CD4^+^ T cells, and activated memory CD4^+^ T cells was higher in the low-risk group, manifesting the protective role in the COAD progression. Naïve B cells are the precursors of functional B cells (Cancro and Tomayko, 2021). B cells were correlated with the better survival, among which B-cell markers could lengthen the survival rate of high-grade tumors (Gunjan et al., 2021). In contrast, the high-risk group had a high proportion of Tregs and M0/M1/M2 macrophages, which is similar with other results (Zhang et al., 2023). Tregs interact with M2 macrophages, which inhibit the antitumor response of colorectal cancer (Lili et al., 2023). Huang et al. (2020) reported that M0 macrophages have tumorigenic effect. These results suggested that the high-risk group has a higher chance of becoming immunosuppressed than the low-risk group.

Our study showed a higher mutation rate of TP53 in the high-risk group. TP53 mutation is the most common somatic mutations in cancer and can be used as a prognostic indicator and target for drug intervention (Olivier et al., 2010; Liebl and Hofmann, 2021). However, our study indicated that a single TP53 mutation does not affect survival, whereas the co-mutation of TP53 and other genes (such as RYR2, SYNE1, TTN, LRP18, and USH2A) significantly shortens the survival time of COAD patients. Previous studies have reported that EGFR and ALK double mutations have been found in the minority of patients with non-small-cell lung cancer (Koivunen et al., 2008), in which patients with double mutations have shorter OS values than patients with EGFR or ALK single mutations (Lou et al., 2016). These findings suggested that the synergistic superposition effect of the co-mutation of TP53 and other genes influenced the prognosis and progress of COAD. In addition, combining TMB values with risk scores was associated with prognosis, indicating that a single TMB value cannot be used as a prognosis factor for COAD, requiring to consider risk scores.

In recent years, the application of immune checkpoint inhibitors in cancer therapy has changed the paradigm of treatment (He et al., 2021). Tumor cells typically escape destruction of cytotoxic T lymphocyte (CTL) by upregulating immune checkpoint ligands such as PD-L1, which can depress lymphocyte activation by binding to complementary receptors (PD-1) on CTL (Klement et al., 2023; Yang et al., 2023). In our study, immune checkpoint-related genes were highly expressed in the high-risk group, suggesting that the high-risk group is suitable for immunosuppressive therapy. TME with highly immunosuppressive properties is a key reason for chemotherapy resistance in cancer patients (Andersen et al., 2021). In our study, ERSG-related scores showed different sensitivity to different chemotherapy drugs, suggesting that the ERSG signature can predict the chemotherapy drug responses. Additionally, the immunotherapeutic predictive efficacy of the ERSG signature was further confirmed from the immunotherapy cohort (anti-PD-L1 in IMvigor210 cohort). These results suggest that our ERSG signature may offer a novel perspective for COAD therapy.

## 5 Conclusion

Taken together, we established the ERSG signature to assess prognosis and immunotherapy sensitivity, helping guide personalized treatment and providing clues to explore the molecular mechanisms between endoplasmic reticulum stress and COAD development.

## Data Availability

Publicly available datasets were analyzed in this study. All datasets used in this study are publicly available on the UCSC Xena platform (https://xenabrowser.net/datapages/) and the GEO (available at: https://www.ncbi.nlm.nih.gov/geo/) database (GSE39582 and GSE200997).

## Supplementary material

SUPPLEMENTARY FIGURE S1 Kaplan–Meier survival analysis identified the prognostic eight ERSGs with *P* < 0.05.

SUPPLEMENTARY FIGURE S2 Kaplan–Meier survival analysis identified the prognostic nine ERSGs with *P* < 0.05.

SUPPLEMENTARY FIGURE S3 Mutation profiles and TMB analyses. **(A)** Mutation profiles of high- (left) and low-risk (right) groups. **(B)** Kaplan–Meier survival analysis of the mutation status of TP53. **(C)** Mutation burden of high- (up) and low-risk (down) groups. **(D)** Comparison of the TMB values between high- and low-risk groups. **(E)** Kaplan–Meier survival analysis of the TMB values.
