# Supplementary material for: Endoplasmic reticulum stress-related prognosis signature characterizes the immune landscape and predicts the prognosis of colon adenocarcinoma
Source: Front Genet. 2025 Apr 1;16:1516232. doi: 10.3389/fgene.2025.1516232 (PMC11996786; doi:10.3389/fgene.2025.1516232)
Supplement: Supplementary file 3 [file Table1.docx]

**Table S1**. The sequences of the q-PCR primers.

| **Gene** | **Forward primer (5’-3’)** | **Reverse primer(5’-3’)** |
| --- | --- | --- |
| DAPK1 | GAGTTTGTCGCTCCTGAGATAGT | GCTTAGTGTCTCCAAGAAATGGG |
| SERPINA1 | GGAGGCTCAGATCCATGAAGG | GGTGTCCCCGAAGTTGACAG |
| HSPA1A | CAGTTCTCAATTTCCTGTGT | TAGTCGTAAGATGGCAGTAT |
| β-actin | GTCATTCCAAATATGAGATGCGT | GCTATCACCTCCCCTGTGTG |
