# Supplementary figures and images for: Endoplasmic reticulum stress-related prognosis signature characterizes the immune landscape and predicts the prognosis of colon adenocarcinoma
Source: Front Genet. 2025 Apr 1;16:1516232. doi: 10.3389/fgene.2025.1516232 (PMC11996786; doi:10.3389/fgene.2025.1516232)

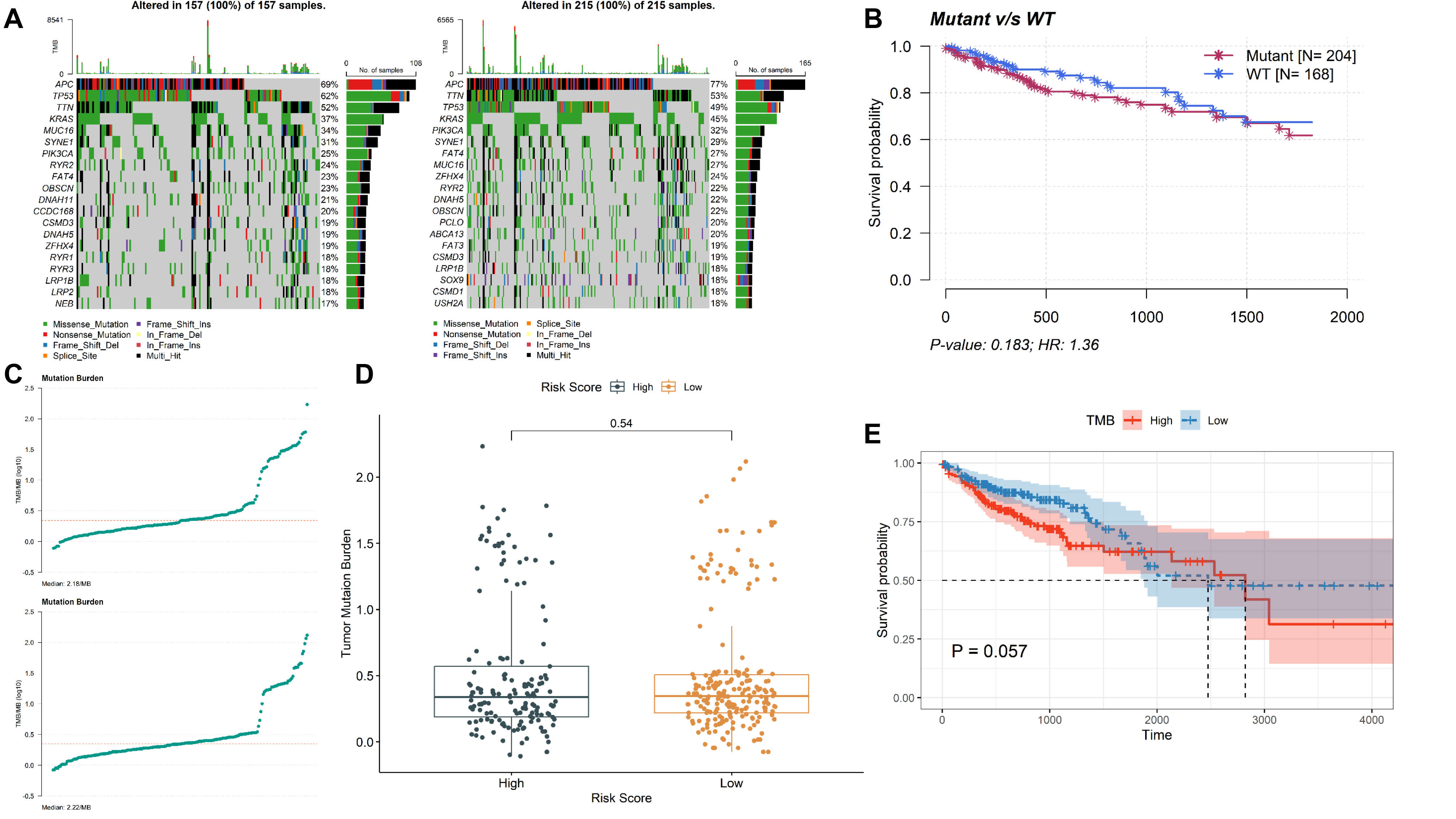

Supplement: Supplementary file 4 [file Image3.tif]

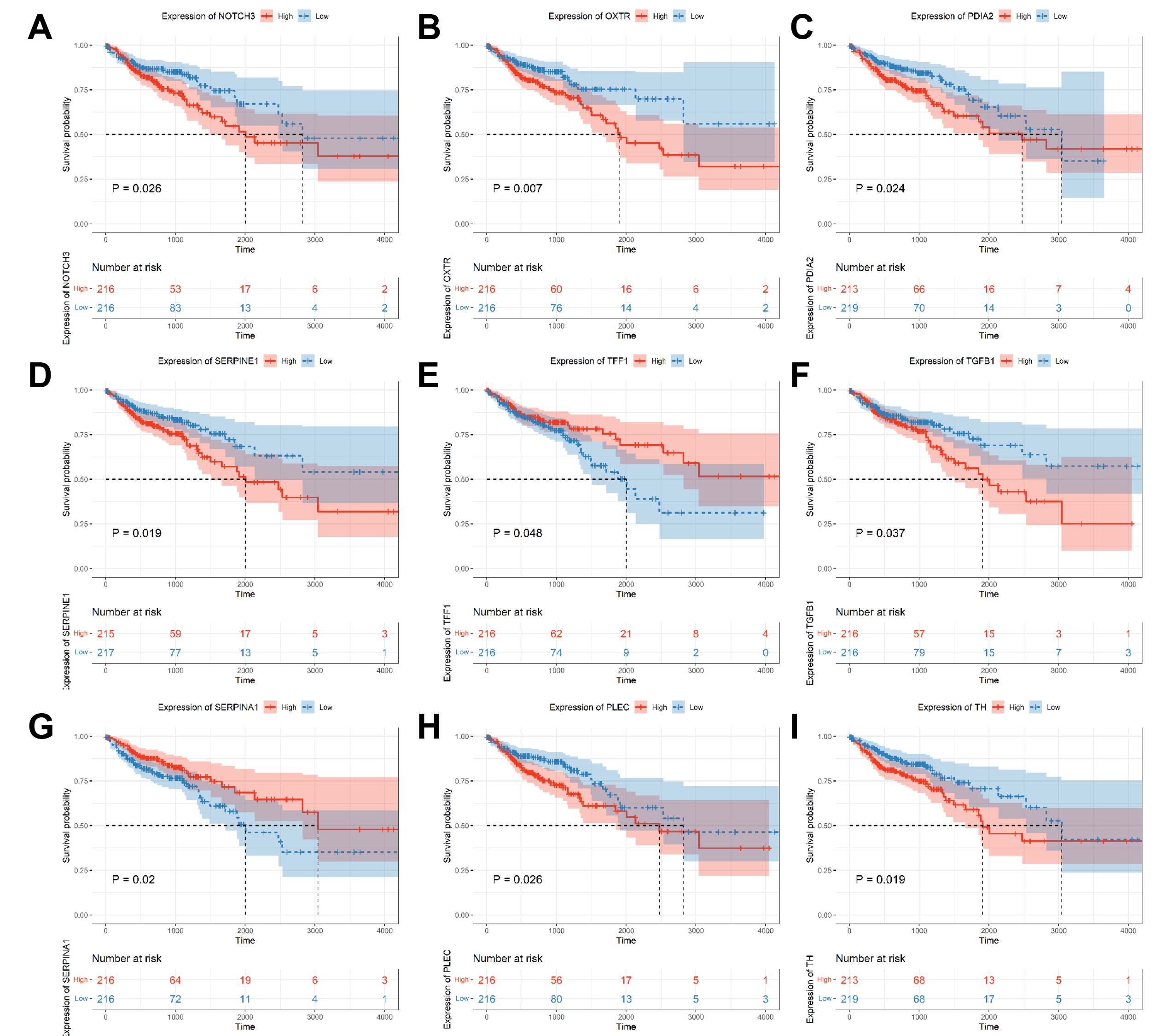

Supplement: Supplementary file 5 [file Image2.tif]

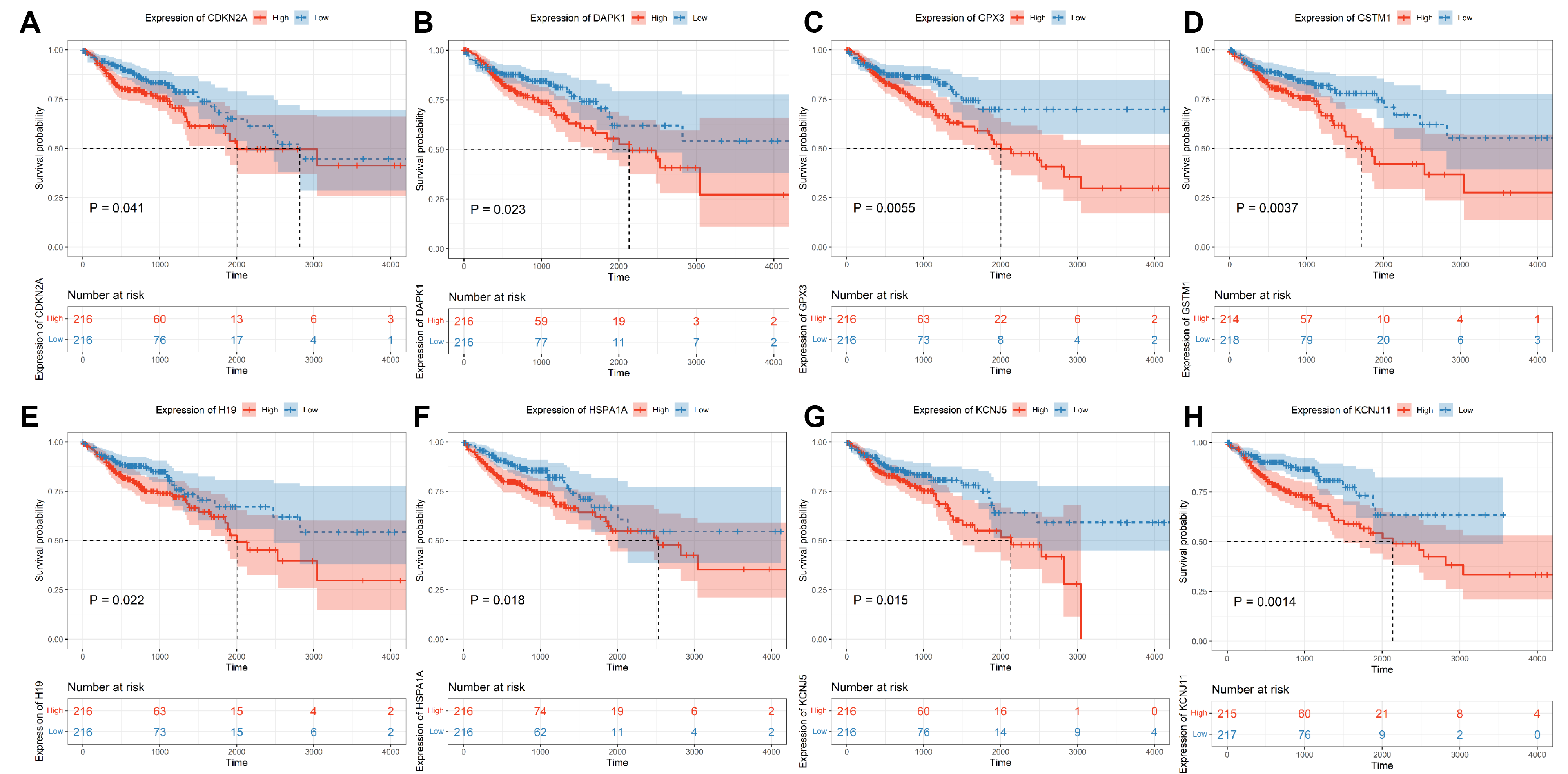

Supplement: Supplementary file 6 [file Image1.tif]
